# Microbial communities of the Mediterranean rocky shore: ecology and biotechnological potential of the sea‐land transition

**DOI:** 10.1111/1751-7915.13475

**Published:** 2019-09-28

**Authors:** Esther Molina‐Menor, Kristie Tanner, Àngela Vidal‐Verdú, Juli Peretó, Manuel Porcar

**Affiliations:** ^1^ Institute for Integrative Systems Biology I^2^SysBio Universitat de València‐CSIC Paterna 46980 Spain; ^2^ Darwin Bioprospecting Excellence S.L. Parc Científic Universitat de València Paterna 46980 Spain; ^3^ Departament de Bioquímica i Biologia Molecular Universitat de València Burjassot 46100 Spain

## Abstract

Microbial communities from harsh environments hold great promise as sources of biotechnologically relevant strains and compounds. In the present work, we have characterized the microorganisms from the supralittoral and splash zone in three different rocky locations of the Western Mediterranean coast, a tough environment characterized by high levels of irradiation and large temperature and salinity fluctuations. We have retrieved a complete view of the ecology and functional aspects of these communities and assessed the biotechnological potential of the cultivable microorganisms. All three locations displayed very similar taxonomic profiles, with the genus *Rubrobacter* and the families *Xenococcaceae*,* Flammeovirgaceae*,* Phyllobacteriaceae*,* Rhodobacteraceae* and *Trueperaceae* being the most abundant taxa; and *Ascomycota* and halotolerant archaea as members of the eukaryotic and archaeal community respectively. In parallel, the culture‐dependent approach yielded a 100‐isolates collection, out of which 12 displayed high antioxidant activities, as evidenced by two *in vitro* (hydrogen peroxide and DPPH) and confirmed *in vivo* with *Caenorhabditis elegans* assays, in which two isolates, CR22 and CR24, resulted in extended survival rates of the nematodes. This work is the first complete characterization of the Mediterranean splash‐zone coastal microbiome, and our results indicate that this microbial niche is home of an extremophilic community that holds biotechnological potential.

## Introduction

The interphase between marine and land environments is an ecologically complex habitat in which selection pressures from both environments can co‐occur. Some of those pressures are high salinity, dehydration, wind and sun exposition, extreme temperature oscillations and mechanical stress associated with seawater splash, often with sand or pebbles, with strong abrasive effects. The aquatic to land transition has been reported to be linked to a narrow gradient in species distribution in function of the distance to the water line, as for example in cyanobacteria in an English lake (Pentecost, 2014). Regarding marine environments, the microbial ecology of rocky shores has previously been analysed (Chan *et al*., 2003; Langenheder and Ragnarsson, 2007; Pinedo *et al*., 2007; Brandes *et al*. 2015), including its links with oil spills and biodegradation (Alonso‐Gutiérrez *et al*., 2009). However, and in contrast with the well‐studied microbial ecology of the intertidal zone (for a review, see Mitra *et al*., 2014), a holistic study on the microbial ecology of the marine supralittoral Mediterranean rocky shore has not been addressed previously.

Harsh, extremophilic environments can be sources of biotechnologically relevant bacteria and therefore hold great promise for the biotechnological industry (Raddadi *et al*., 2015). For example, extremophilic microorganisms can yield enzymes such as lipases and esterases that can be used under a wide range of conditions and may have relevant applications in the food, detergent and biofuel industries (Fuciños *et al*., 2012). There are many other examples of biotechnologically relevant microorganisms from extreme environments, including the well‐known case of *Thermus aquaticus*, which produces the widely used *Taq* polymerase; or the hyperthermophilic biofuel‐producing archaea that live in deep‐sea hydrothermal vents (Chien *et al*., 1976; Nishimura and Sako, 2009).

The present study focuses on the microorganisms that inhabit the rocky areas of the supralittoral zone (the area just above the tide line that is subjected regularly to splash but is not permanently underwater) of the Mediterranean coast. Surface‐associated microbial communities that are sun‐exposed are often rich in microorganisms that produce pigments, including carotenoids (Dorado‐Morales *et al*., 2015; Kumar *et al*., 2015; : Shindo and Misawa, 2014; Tanner *et al*., 2017). These pigments play a key role in radiation tolerance (Tian and Hua, 2010; Klindworth *et al*., 2013; Sandmann, 2015; Tanner *et al*., 2018), and they are valuable for the food, pharmacological and cosmetic industries as colourants, antioxidants and protectors against solar radiation respectively (Sandmann, 2015). Therefore, we hypothesized that rough conditions of the supratidal zone may be associated with the presence of biotechnologically relevant microbial taxa. From this hypothesis, we have, in the present work, compared three different supralittoral coastal locations of the Mediterranean West coast and combined culturing techniques and high throughput sequencing data (16S rRNA amplicon and metagenomic sequencing) in order to shed light on the taxonomic composition of these communities, and to explore the biotechnological potential of the culturable strains.

## Results

### High‐throughput 16S rRNA analysis

**Figure 1:**
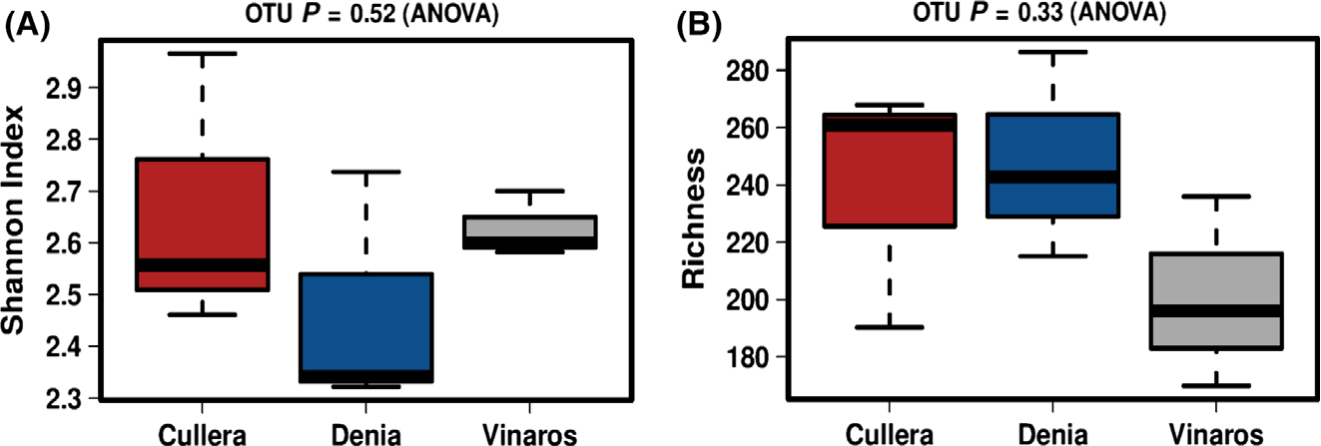
Box plots showing the values of alpha diversity indexes in the sampled locations on the Mediterranean rocky‐shore. (A) Observed richness at OTU level (number of OTUs). (B) Shannon index of diversity.

High‐throughput 16S rRNA sequencing of the samples revealed that, based on the comparison of the richness value (number of different species; Fig. 1A) and the diversity (Shannon index; Fig. 1B), the alpha diversity was not significantly different among the locations. Moreover, the shape of the rarefaction curve at OTU level (Operational Taxonomic Unit) showed that the sequences covered the majority of taxa present in the samples (Fig. S1).

**Figure 2:**
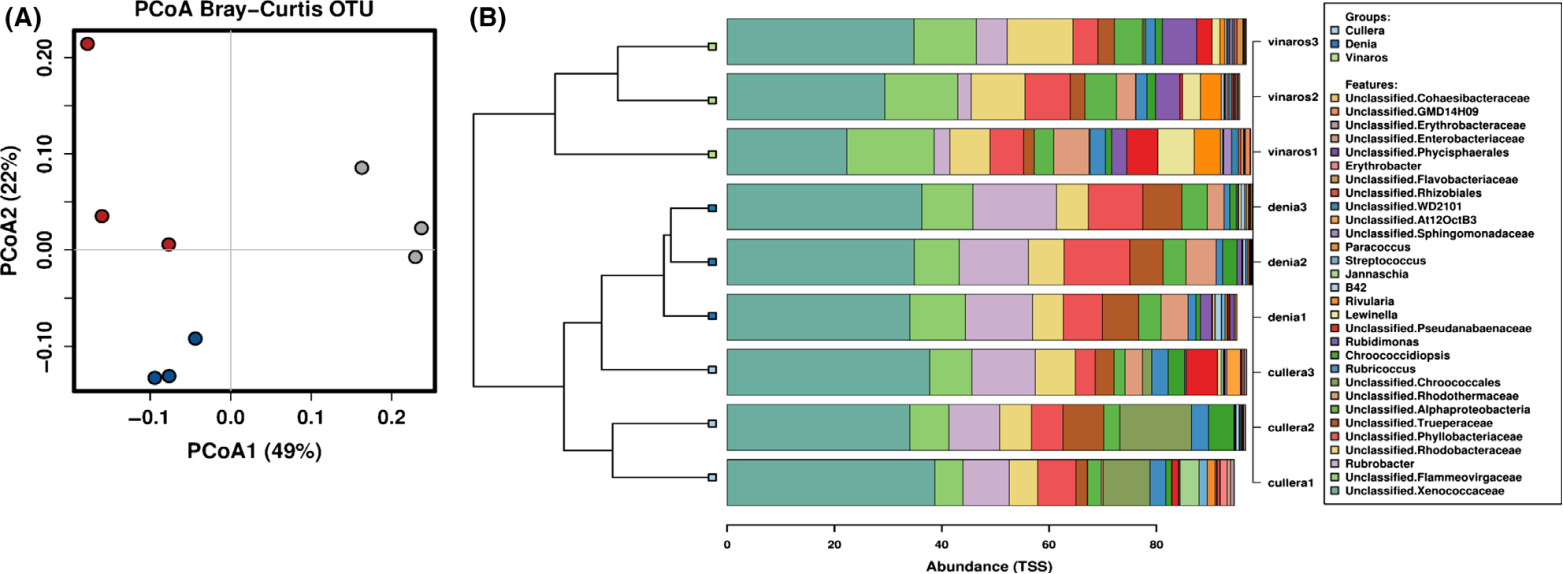
(A) Principal coordinates analysis (PCoA) based on Bray–Curtis distances between OTUs in bacterial communities of three different locations. (B) Clustered‐Barchart showing the top 30 most abundant genera in terms of relative abundance.

However, the composition of the bacterial communities varied depending on the location, as represented in the Principal Coordinates Analysis (PCoA; Fig. 2A). Samples from Dénia showed the highest intragroup homogeneity, whereas samples from Vinaròs and Cullera displayed higher differences between replicates. Nevertheless, samples from all three locations could be distinguished in the plot. The variability explained by both axes is high enough to conclude that the microbial communities among the three locations are different. Moreover, the representation of the relative abundances (TSS) of the top 30 most abundant genera showed that the microbial composition was generally similar along the locations (Fig. 2B), although some taxa such as the genus *Rubrobacter* in Vinaròs or the genus *Rubricoccus* in Dénia allowed the differentiation of specific regions (Table 1). Eleven out of the 30 most abundant genus were significantly different at least in one location. A list of the 30 more significantly different genus is shown in (Table S1). The original data have been deposited with the NCBI SRA accession number PRJNA556782.

**Table 1:** Top 30 most abundant genera and *P*‐values for the One‐Way ANOVA statistical analysis of their distributions among the three sampled locations

Taxa	*P* labelA	*P* (Tukeys) Dénia‐Cullera	*P* (Tukeys) Vinaròs‐Cullera	*P* (Tukeys) Vinaròs‐Dénia
*Rubrobacter*	0.0011*	0.083	0.0096*	0.00091*
*Rubricoccus*	0.0018*	0.0014*	0.057	0.026*
Unclassified *Flammeovirgaceae*	0.006*	0.22	0.0051*	0.04*
*Rubidimonas*	0.0075*	0.58	0.0078*	0.024*
Unclassified *Erythrobacteraceae*	0.0085*	0.011*	0.018*	0.9
Unclassified *Alphaproteobacteria*	0.019*	0.052	0.02*	0.71
Unclassified *Cohaesibacteraceae*	0.021*	0.021*	0.062	0.66
*Rivularia*	0.026*	1	0.04*	0.038*
Unclassified *Rhodobacteraceae*	0.037*	1	0.058	0.052
Unclassified WD2101	0.039*	1	0.06	0.054
Unclassified *Chroococcales*	0.045*	0.063	0.068	1
*Lewinella*	0.052	1	0.073	0.075
Unclassified *Trueperaceae*	0.066	0.29	0.45	0.056
Unclassified *Phyllobacteriaceae*	0.091	0.094	0.87	0.18
Unclassified *Xenococcaceae*	0.1	0.85	0.1	0.21
Unclassified GMD14H09	0.12	1	0.17	0.16
Unclassified *Sphingomonadaceae*	0.13	0.88	0.24	0.13
Unclassified *Flavobacteriaceae*	0.13	1	0.16	0.17
B42	0.18	0.19	0.95	0.28
Unclassified *Rhodothermaceae*	0.24	0.23	0.47	0.82
Unclassified *Rhizobiales*	0.25	0.72	0.55	0.22
*Chroococcidiopsis*	0.28	0.4	0.3	0.97
*Erythrobacter*	0.28	0.29	0.42	0.95
Unclassified *Phycisphaerales*	0.32	0.29	0.66	0.73
Unclassified *Pseudanabaenaceae*	0.34	0.51	0.93	0.33
*Jannaschia*	0.39	0.44	0.47	1
*Paracoccus*	0.44	0.78	0.41	0.78
Unclassified At12OctB3	0.46	0.49	0.55	0.99
*Streptococcus*	0.54	0.91	0.52	0.76
Unclassified *Enterobacteriaceae*	0.77	0.91	0.75	0.95

Global *P*‐values and *P*‐values for the comparison by pairs is shown. Significant results are marked by an asterisk.

### Shotgun metagenomic analysis

The three locations exhibited similar taxonomic profiles according to the metagenomics analysis. The most abundant bacterial phyla were the same ones observed with high‐throughput 16S rRNA sequencing, with *Cyanobacteria* being the most abundant in all three locations. Moreover, other taxa, such as the families *Rhodobacteraceae*,* Flammeovirgaceae*,* Trueperaceae* and the genus *Rubrobacter*, belonging to the phyla *Proteobacteria*,* Bacteroidetes*,* Deinococcus‐Thermus* and *Actinobacteria* respectively, were also detected (Figs S2A, S3A and S4A). Metagenomic sequencing allowed the identification of abundant taxa in the *Cyanobacteria* phylum, including the genera *Staniera, Pleurocapsa*,* Myxosarcina* and *Xenococcus*, in contrast to the high‐throughput 16S rRNA, which mainly showed unclassified *Xennococcaceae* taxa.

Archaeal and eukaryotic communities proved very diverse, with a high number of salt‐adapted microorganisms in the former and a large fraction of *Ascomycota* in the latter (Figs S2, S3, S4B and C). Salt‐adapted archaea included members of *Halococcus*,* Halobacteriaceae* (*Haladaptatus* and *Halalkalicoccus*), *Haloarculaceae*,* Haloferaceae*,* Halorubraceae* and *Natrialbaceae* families, as well as methanogenic archaea (members of the *Methanosarcinaceae* family; Figs S2B, S3B and S4B). Among the diversity of *Ascomycota*, the most abundant taxa were *Glonium stellatum*,* Cenococcum geophilum*,* Coniosporium apollinis* and *Lepidopterella palustris* (Figs S2C, S3C and S4C).

**Figure 3:**
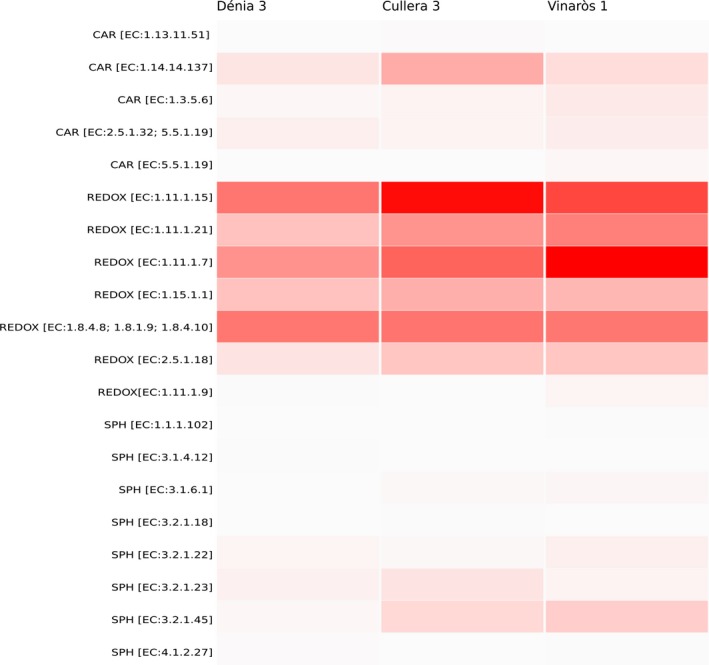
Heatmap representing the functional analysis carried out through metagenomics sequencing. Enzymes related to carotenoid biosynthesis (CAR), oxidative stress (REDOX) and sphingolipid biosynthesis (SPH) are shown in the *Y*‐axis.

The functional analysis of the samples revealed a high representation of enzymes related to oxidative stress, being peroxiredoxin (EC 1.11.1.15) and peroxidase (EC 1.11.1.7) the most abundant activities, and displaying the highest values in Cullera and Vinaròs respectively. Thioredoxin‐related enzymatic activities (EC 1.8.4.8; EC 1.8.1.9; EC 1.8.4.10) were homogeneously represented in all three samples, as well as superoxide dismutase (EC 1.15.1.1). Other enzymes such as glutathione transferase (EC 2.5.1.18) or glutathione peroxidase (EC 1.11.1.9) varied among locations, with the former being more represented in Vinaròs and Cullera than in Dénia, and the latter being more abundant in Vinaròs. Among the genes related to carotenoid biosynthetic routes, the abscisic acid 8′‐hydroxylase (EC 1.14.14.137) was particularly represented in Cullera, whereas sphingolipid‐related genes such as glucosylceramidase proved to be frequent in Vinaròs (EC 3.2.1.45; Fig. 3). The original data have been deposited with the NCBI SRA accession number PRJNA556786.

### Strain collection and identification

Culturing the samples on LB and Marine Agar yielded a large diversity of colonies in terms of colour, shape and morphology. A total of 100 strains were isolated and named with a code, after the location (C: Cullera, D: Dénia, V: Vinaròs) and the origin (M: Marine water, R: Rock surface). In our conditions, there was no significant fungal growth in any of the samples. The colonies observed on Marine Agar displayed the widest range of colours (wine‐red, red, pink and orange, among others) in comparison with the ones observed on LB media, which were mostly yellowish and cream‐coloured. Due to the known relation between the presence of pigments and antioxidant power, the main criterion for colony selection was the colour (Pawar *et al*., 2015).

A collection of the 100 selected isolates in pure culture was established. A total of 34 isolates were initially identified through colony PCR and 16S rRNA Sanger sequencing. Although an initial step of incubation at 100°C was added to the PCR protocol of the isolates whose amplification had failed, some remained non‐identified and therefore their total DNA was extracted to repeat the PCR. Finally, 56 of the isolates remained unidentified. Among the identified isolates, there were many *Bacillus* spp. (*B. oleronius*,* B. licheniformis*,* B. marisflavi*,* B. salsus* and *B. altitudinis*) and *Halobacillus* spp. (*H. trueperi* and *H. faecis*) as well as other species such as *Micrococcus antarcticus*,* Micrococcus luteus*,* Staphylococcus pasteuri*,* Vibrio tubiashii* and *Virgibacillus halodenitrificans* (Table S2).

### Antioxidant activity

In order to select and establish a collection of isolates with antioxidant properties, a high‐throughput screening of the 100 isolates was performed by growing them on solid media containing H_2_O_2_. *Planomicrobium glaciei* and *E. coli* JM109 were used as positive and negative controls respectively. Strain JM109, with no known reports of antioxidant effect, exhibited a weak growth in the first (OD_600_ 1) and, sometimes, second dilution (OD_600_ 10^−1^). This led us to the criterion to consider positive antioxidant producers those strains able to grow on H_2_O_2_‐containing plates at least up to threefold dilutions (OD_600_ 10^−2^). A total of 12 isolates were thus selected (Table 2) based on their ability to grow on 1 mM H_2_O_2_ plates as described above.

**Table 2:** List of selected isolates, percentage of identity with the closest type strain, sequence similarity and results obtained in the H_2_O_2_ assay

Sample	Closest type strain	%	H_2_O_2_ Assay (dilution at which the isolate remains viable)
CR10	*Micrococcus luteus* (CP001628)	99.77	3
CR17	*Virgibacillus halodenitrificans* (AY543169)	99.58	7
CR21	Non‐identified	–	4
CR22	*Virgibacillus halodenitrificans* (AY543169)	99.37	4
CR24	*Halobacillus trueperi* (AJ310349)	98.31	6
CR28	*Virgibacillus halodenitrificans* (AY543169)	100	6
CR37	*Bacillus marisflavi* (LGUE01000011)	100	4
CR44	Non‐identified	–	3
CR67	*Bacillus oleronius* (X82492)	97.32	4
DM10	Non‐identified	–	3
DR12	Non‐identified	–	3
VR1	*Bacillus altitudinis* (ASJC011000029)	100	6
VR2	*Micrococcus luteus* (CP001628)	99.35	3
Positive control	*P. glaciei*		8
Negative control	*E. coli* (JM109)		1

DPPH‐based assays are widely used to detect and quantify the antioxidant power of plants or bacterial extracts. These assays are based on the decrease of DPPH absorbance at 517 nm in presence of antioxidant factors. The oxidative stress‐resistant isolates selected from the H_2_O_2_ assay (shown in Table 2) were further tested using this method. CR17, CR21 and CR57 could not be tested due to poor growth in liquid culture, which made it impossible to obtain a concentrated extract, prepared as described in Experimental Procedures. 16S rRNA sequences were compared using NCBI BLAST tool. Isolates CR10‐VR2 and CR22‐CR28 were 100% identical in their 16S rRNA sequence, and therefore only one of them was selected for further assays (CR10 and CR22 respectively).

**Figure 4:**
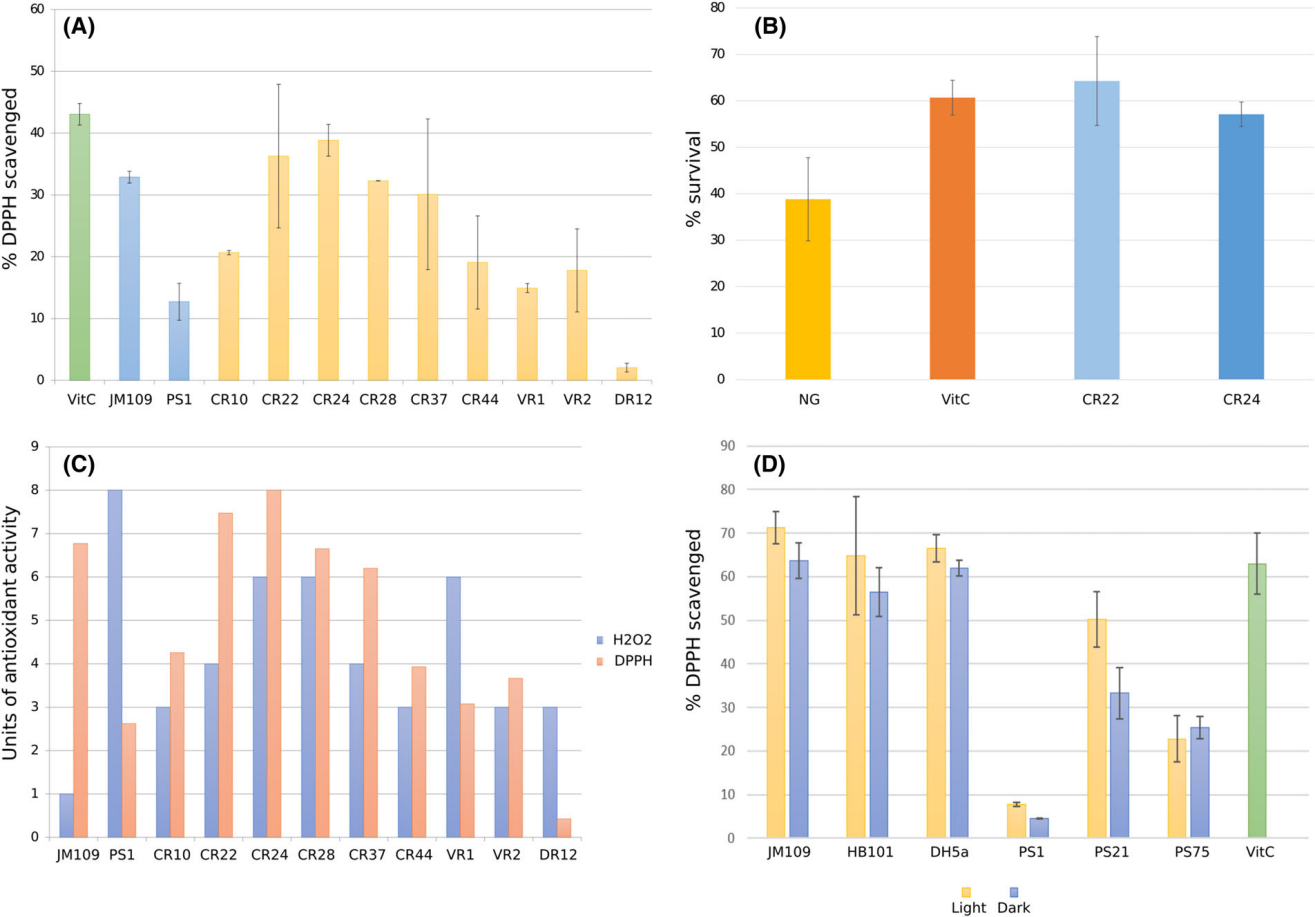
(A) Antioxidant activity as measured through DPPH assay as described in EP. Absorbance was measured at 517 nm after 30 min of incubation with DPPH 50 μM. DPPH scavenged (%) is represented in *Y*‐axis. VitC, vitamin C (0.5 μg ml^−1^ solution). (B) Antioxidant activity *in vivo* (using the model organism *C. elegans*). *Y*‐axis indicates percentage of surviving worms after 5 h of incubation under oxidative stress (H_2_O_2_). Worms were treated with either a control diet (NG), a diet supplemented with the known antioxidant vitamin C as a positive control (VitC), or a diet supplemented with the selected strains CR22 and CR24. (C) Comparative analysis of the results obtained with H_2_O_2_ and DPPH assays. Values in *Y*‐axis are normalized with respect to the highest value obtained in both assays. (D) DPPH assay with positive and negative controls. Absorbance was measured at 517 nm after 30 min of incubation with DPPH 50 μM. DPPH scavenged percentage is represented in *Y*‐axis. VitC, Vitamin C, 0.5 μg ml^−1^ solution. Light and dark conditions are represented.

The test resulted in a general decrease in absorbance in all the samples, suggesting that the extracts were able to scavenge the DPPH. The isolates that proved more effective as antioxidants were CR22, CR24 and CR28, with values of scavenged DPPH over 30% (Fig. 4A). DR12 displayed low DPPH scavenging values maybe due to failure of the pigment extraction. Surprisingly, the control samples *P. glaciei* and JM109 did not display the expected effect. A set of three strains that had previously shown a protective effect against oxidative stress in a *Caenorhabditis elegans* model and a set of three *E. coli* strains (JM109, HB101 and DH5α) were also tested (Fig. 4D).

The two strains with the best results in the *in vitro* assays (CR22 and CR24) were selected for further *in vivo* antioxidant assays in the model organism *C. elegans,* where both proved able to display an important antioxidant activity (Fig. 4B). Nematodes subjected to oxidative stress after being treated with isolates CR22 and CR24 displayed survival rates higher than the untreated worms and similar to those observed in the worms treated with vitamin C (survival rates of around 55%–65%).

## Discussion

We report here, for the first time, and by using culture‐dependent and independent (NGS) techniques, the microbiomes of the rocky‐coastal surface of the supralittoral zone in three regions on the Mediterranean western coast. The three sampled sites, covering a coast line of about 260 km, displayed remarkably similar taxonomic profiles in terms of richness and microbial diversity, but still could perfectly be differentiated thanks to the significant difference in abundances of specific taxa, which suggest that the microbial composition of the Mediterranean supratidal zone, at least in eastern Spain, is stable but not identical within rocky locations. The studied communities were particularly dominated by bacterial strains previously described as thermophilic, halotolerant or radioresistant, such as the species within the genus *Rubrobacter* (Jurado *et al*., 2012), and pigmented isolates, as is the case of species within the *Flameovirgaceae* family, like *Tunicatimonas pelagia* and *Porifericola rhodea* (Yoon *et al*., 2011, 2012).

*Truepera radiovictrix*, characterized by an optimum growth temperature of 50°C and an extreme resistance to ionizing radiation, was first isolated from a hot spring in a geothermal area close to the Azores (Albuquerque *et al*., 2005; Ivanova *et al*., 2011). Moreover, the *Truepera* genus has been previously found in Lake Lucero Playa (New Mexico, USA), a particularly hostile environment as the lake dries periodically (Sirisena *et al*., 2018). This is, to the best of our knowledge, the first report of sea‐inhabiting *Truepera* in a non‐thermal environment, and it is tempting to hypothesize that the genus *Truepera* might have a similar ecological niche (radiation‐ and desiccation‐resistance) than *Deinococcus*, but in saline environments, as a consequence of both its radiation resistance and halotolerance (Albuquerque *et al*., 2005).

Shotgun metagenomic analysis confirmed the similarity between the communities of the three sampled locations, as discussed above from the high‐throughput 16S rRNA results, particularly at higher taxonomic (i.e. family) levels. Nevertheless, the results at lower taxonomic levels varied considerably among sequencing techniques. One of the largest differences at the species level was observed within the Cyanobacterial group. In particular, high‐throughput 16S rRNA revealed a large abundance of *Xennococcaceae*, whereas shotgun metagenomic sequencing revealed a more diverse population including members of *Pleurocapsa*,* Myxosarcina, Stanieria* and *Xenococcus*, as previously reported for marine environments (Burns *et al*., 2004; Alex *et al*., 2012; Yu *et al*., 2015; Brito *et al*., 2017).

The eukaryotic fraction of the samples was mainly composed of *Ascomycota*, such as *Glonium stellatum*. The genus *Glonium* includes saprophytic *Dothideomycetes* that produce darkly pigmented apothecia, which could contribute to the dark colour of the sampled rocks (Spatafora *et al*., 2012). Other species detected in the samples included as follows: *Cenococcum geophilum*, an ectomycorrhizal fungus previously described in coastal forest soils (Matsuda *et al*., 2015) and previously demonstrated to grow at up to 100 mM of NaCl (Obase *et al*., 2010); *Coniosporium apollinis*, a rock‐inhabiting fungi previously isolated from the Mediterranean basin (Sterflinger *et al*., 1997); and *Lepidopterella palustris*, typically a freshwater fungus (Shearer *et al*., 2009), with this being, to the best of our knowledge, the first description of this species in a salt water habitat.

Taken together, the results obtained from both high‐throughput 16S rRNA and metagenomic sequencing suggest that the sampled communities are composed of a diverse array of fungi (mainly belonging to the phylum Ascomycota), cyanobacteria (mainly *S. cyanosphaera* and *Pleurocapsa* spp., but also *Myxosarcina* spp. and *Xenococcus* spp.) and salt‐adapted archaea, which remain rather stable among the three different sampled locations.

From the functional point of view, metagenomics sequencing showed abundance of enzymes involved in oxidative stress, mainly peroxidase, peroxiredoxin and thioredoxin, but also catalase and glutathione transferase. In contrast with this, enzymes involved in carotenoid or sphingolipid biosynthesis, which also play a role in the protection against oxidative stress, were less abundant and varied among locations, being abscisic acid 8′‐hydroxylase (EC 1.14.14.137) in Cullera and glucosylceramidase (EC 3.2.1.45) in Vinaròs the ones with the highest values.

From the collection of cultured microorganisms, a total of 12 isolates were selected for their high antioxidant activity as measured by the oxidative stress assay performed with H_2_O_2_. Of those, *M. luteus* has been reported to encode genes related to resistance and tolerance to oxidative stress (superoxide dismutase and NADP reductase; Lafi *et al*., 2017). The DPPH assay was performed to dismiss false positives through the H_2_O_2_ assay. In general, the results correlated well with the ones previously observed in the H_2_O_2_ assay. It is important to note that, although DR12 displayed low scavenging in the DPPH assay, the extraction of pigments from this isolate was sub‐optimal, since the pellet remained pink‐coloured after the extraction process. Surprisingly, the control samples *P. glaciei* and JM109 did not display the expected effect in terms of antioxidant activity. On one hand, *P. glaciei* was expected to be one of the most antioxidant isolates, as its antioxidant activity was demonstrated in previous *in vivo* assays in *C. elegans* (Tanner *et al*., 2019) and in the H_2_O_2_ assay. Nevertheless, it was the worst strain in terms of DPPH scavenging. On the other hand, *E. coli * JM109, with no previous reports on antioxidant activity, resulted in high DPPH scavenging. This raises concerns on the suitability of DPPH‐methods in bioprospecting for the determination of antioxidant activity and highlights the importance of using several alternative methods as the best option to have a proxy of the *in vivo* antioxidant effects. Nevertheless, the *in vivo* antioxidant assay performed in *C. elegans* allowed to confirm the antioxidant activity detected in the DPPH and H_2_O_2_ tests. Specifically, CR22 and CR24 displayed an antioxidant activity similar to the one observed in Vitamin C (Fig. 4B).

In general, though, the correlation between both methods was good, as the isolates with higher survival in the presence of H_2_O_2_ also displayed higher DPPH‐scavenging ability (Fig. 4C). Nevertheless, there were some isolates that displayed different results depending on the method, in particular VR1 and CR37. Differences in VR1 could be the result of catalase activity, which may have enhanced its growth on the H_2_O_2_‐supplemented plates. On the contrary, differences between both methods for CR37 could be caused by a deficient growth in solid medium. Once again, these results highlight the limitation of using a single screening technique for the selection of microbial strains with antioxidant activities.

A collection of both positive and negative controls (in terms of theoretical antioxidant activity) were tested using both assays (H_2_O_2_ and DPPH). PS1, PS21 and PS75 (*P. glaciei* 423, 97.38% ID; *Rhodobacter maris* JA276, 98.89% ID; and *Bacillus megaterium* NBRC 15308, 100% ID respectively) were the three control strains selected, all of them recovered from solar panels and previously tested in *C. elegans* for *in vivo* protection against oxidative stress (Tanner *et al*., 2019). Three different strains of *E. coli* were chosen as negative controls (JM109, BH101, DH5α). For the DPPH assay, the isolates were grown under both light and dark conditions, in order to determine whether the light had a negative impact on the production of pigments or other antioxidant factors, as it is known that many pigments, particularly carotenoids, are prone to photodegradation (Boon *et al*., 2010). For the *E. coli* strains, no significant differences were observed between growth in dark and light conditions, whereas PS21 proved very sensitive to light (Fig. 4D). Moreover, the scavenging effect of the JM109 strain was also observed in the other two *E. coli* strains, confirming that the extracts obtained from *E. coli* contain compounds that are indeed able to react with DPPH. Even though *R. maris* and *B. megaterium* displayed better antioxidant properties than *P. glaciei*, which was again comparable to the negative control of methanol, they yielded lower DPPH‐based activity than *E. coli* strains.

The biotechnological potential of extremophiles is well known, and saline environments are no exception to this rule (de Lourdes Moreno *et al*., 2013). However, and in contrast with the well‐studied intertidal zone (Mitra *et al*., 2014), the supralittoral zone has been poorly studied to date. Interestingly, this zone experiences much higher selection pressures than the intertidal zone since while the intertidal zone is basically a marine environment which is only transiently and partially exposed to land conditions, the supralittoral zone forces organisms to adapt to a sea/land intermediate habitat where both marine and land stresses are present.

This work is the first holistic (using culture‐dependent, culture‐independent and biological activity assays) approach studying the microbial ecology and biotechnological potential, in terms of antioxidant properties, of the supralittoral zone of the Mediterranean rocky shore. Our results suggest that the western coastline of the Mediterranean Sea harbours a stable microbial community that is conserved among different locations, with cyanobacteria as the majoritarian bacterial taxon, followed by members of the *Flameovirgaceae* family and members of the *Rubrobacter* genus, as well as eukaryotic and archaeal members, such *Ascomycota* and halotolerant archaea. Furthermore, *in vitro* and *in vivo* assays demonstrate that this environment is a potential source of microorganisms with antioxidant activities that could hold potential for a wide range of applications in the food, cosmetic or pharmacological industries.

## Experimental procedures

### Sampling

Samples were collected from three different locations on the Mediterranean Western coast, in Eastern Spain: Vinaròs (Castelló), Cullera (València) and Dénia (Alacant). Three samples of dark‐stained rock, at least two metres apart from each other and thus considered as biological replicates, were collected from the supralittoral (splash) zone of each location by scraping the surface with a sterile blade. Samples of the adjacent marine water were also taken, and both types of samples (scrapped rock and sea water samples) were separately stored in Falcon tubes in 15% glycerol, transported to the laboratory on ice and then stored at −20°C until required.

### High‐throughput rRNA and metagenomic sequencing

Total DNA was isolated from the samples with the PowerSoil DNA Isolation kit (MO BIO laboratories, Carlsbad, CA, USA) following the manufacturer's instructions. The quantity and quality of the isolated DNA was assessed using a Nanodrop‐100 Spectrophotometer (Thermo Scientific, Wilmington, DE, USA) and purified DNA samples were sequenced by Life Sequencing SL (València, Spain). On one hand, the hypervariable V3‐V4 regions of the 16S rRNA gene was amplified as described by Klindworth *et al*. (2013) and sequenced on the high‐throughput NextSeq 500 (Illumina) platform. Greengenes database was used for the taxonomic analysis. The statistical analysis was carried out with Calypso web tool (version 8.84; http://cgenome.net). The statistical comparison of the relative abundances between locations at the genus level was calculated through One‐Way Anova test (Tables 1 and S1). Richness and Shannon index box plots, PCoA, relative abundances clustering and rarefaction curve were also constructed with Calypso.

On the other hand, shotgun metagenomic sequencing was performed on the NextSeq500 Illumina platform, with paired‐end sequences and reads of 150 base pairs. The obtained sequences were filtered by using ‘BBtools’ version 37.28 (https://jgi.doe.gov/data-and-tools/bbtools/) in order to avoid ends holding quality values under the Q20 standards. Lectures coming from human contamination were also dismissed by mapping them against the reference human genome (GRCh37d5) version 0.7.15. Assembly was carried out with ‘SPAdes’ (Bankevich *et al*., 2012) version 3.9. ORFs prediction was carried out by ‘MegaGeneMArk’ (Zhu *et al*., 2010) version 3.38 and rRNA prediction, by ‘RNAmmer’ (Lagesen *et al*., 2007) version 1.2. Functional annotation of the predicted CDS was carried out with *BLAST2go* (Conesa *et al*., 2005) version 4.1.9.

The Clustergrammer on‐line software (Fernández *et al*., 2017) was used for the functional analysis heatmap construction, by using a correlation type distance and average linkage.

### Isolation and identification of bacterial strains

Three different growth media were used for this study: Lysogenic Broth (LB, composition in g l^−1^: 10 tryptone, 10 NaCl, 5.0 yeast extract, 15 agar); Reasoner's 2A agar (R2A, composition in g l^−1^: peptone 0.5, casaminoacids 0.5, yeast extract 0.5, dextrose 0.5, soluble starch 0.5, K_2_HPO_4_ 0.3, MgSO_4_ 0.05, sodium pyruvate 0.3, 15 agar); and Marine Agar (composition in g l^−1^: peptone 5.0, yeast extract 1.0, ferric citrate 0.1, NaCl 19.45, MgCl_2_ 5.9, Na_2_SO_4_ 3.24, CaCl_2_ 1.8, KCl 0.55, NaHCO_3_ 0.16, KBr 0.08, SrCl_2_ 0.034, H_3_BO_3_ 0.022, Na_4_O_4_Si 0.004, NaF 0.024, NH_4_NO_3_ 0.0016, Na_2_HPO_4_ 0.008, 15 agar). The scraped rock samples were homogenized in the Falcon tube by vigorously mixing with a vortex, and serial dilutions were cultured on the different media and incubated at room temperature for 7 days. Marine water samples were also cultured in the same conditions. After 1 week of incubation, individual colonies were selected based on colony pigmentation and isolated by independent re‐streaking on fresh medium. Pure cultures were then cryo‐preserved at −80°C in 20% glycerol (vol:vol) until required.

Colony PCR and, were needed, DNA extracts of each of the isolated strains, were used for taxonomic identification through 16S rRNA gene sequencing using universal primers 28F (5′‐GAG TTT GAT CNT GGC TCA G‐3′) and 519R (5′‐GTN TTA CNG CGG CKG CTG‐3′). Colony PCR was performed with an initial step of incubation at 95°C for 5 min to lyse cells followed by PCR amplification (30 cycles of 30 s at 95°C, 30 s at 54°C, 30 s at 72°C, followed by 10 min at 72°C). The DNA extraction was done following the Latorre *et al*. (1986) protocol. Amplifications were verified by electrophoresis in a 0.8% agarose gel and then amplicons were precipitated overnight in isopropanol 1:1 (vol:vol) and potassium acetate 1:10 (vol:vol; 3 M, pH 5). DNA pellets were washed with 70% ethanol and resuspended in 30 μl Milli‐Q water. BigDye^®^ Terminator v3.1 Cycle Sequencing Kit (Applied Biosystems, Carlsbad, CA, USA) was used to tag amplicons, which were sequenced with the Sanger method by the Sequencing Service (SCSIE) of the University of Valencia (Spain). All sequences were manually edited with Pregap4 (Staden Package, 2002) to eliminate low‐quality base calls, and final sequences were compared by EzBioCloud 16S tool (https://sourceforge.net/projects/staden/).

### Antioxidant activity

#### Hydrogen peroxide assay

The collection of isolates was initially screened for antioxidant activity by applying oxidative stress to the isolated colonies through the addition of hydrogen peroxide (H_2_O_2_) to the growth medium. In order to do so, isolates were grown on solid media for 4 days or until reaching enough biomass. Then, the optical density at 600 nm (OD_600_) was measured, adjusted to a value of 1, and serial dilutions prepared up to seven times fold. Two microlitres of each dilution were placed on a LB or Marine Agar place, to which 1 mM H_2_O_2_ had been previously added. The plates were incubated at room temperature and in the dark to avoid degradation of the H_2_O_2_, and results were recorded after two, four and six days. Two strains were used as controls for the assay: PS1 (*Planomicrobium glaciei* 423, 97.38% ID) and *Escherichia coli* JM109 as a positive and negative control for antioxidant activity respectively. *Planomicrobium glaciei* is a pigmented microorganism whose antioxidant activity has previously been reported *in vivo* using a *Caenorhabditis elegans* model (Tanner *et al*., 2019).

#### DPPH assay

Since the H_2_O_2_ assay can result in false‐positive results due to catalase activity, a second assay using 2,2‐diphenyl‐1‐picrylhydrazyl (DPPH) was performed to dismiss false positives in the H_2_O_2_ assay and to confirm the antioxidant activity of the selected strains (the ones with the best antioxidant activity according to the previous assay). Pigments were extracted from the isolates based on the protocols described by Brand‐Williams *et al*. (1995), von Gadow *et al*. (1997) and Su *et al*. (2015), with the modifications suggested by Sharma and Bhat (2009). Briefly, the isolates were grown overnight in liquid LB medium and OD_600_ was measured and normalized at a value of 1.2. Cells were then harvested by centrifugation at  11,300 *g* for 3.5 min, and the pellets resuspended in 500 μL of methanol, vigorously vortexed and sonicated for 5 min (Ultrasonic bath XUBA1, Grant Instruments, Royston, UK). The supernatant was collected after centrifugation at 11,300 *g* for 3 min and kept in the dark until the assay was performed. The extraction was repeated as described until a colourless pellet was obtained.

For the DPPH assay, 600 μl of the extract in methanol were mixed with 400 μl of DPPH solution (50 μM in methanol) and incubated for 30 min in the dark. The negative control sample consisted of DPPH mixed with methanol. Absorbance was measured at 517 nm (Ultrospec 200 UV/V Visible Spectrophotometer, Pharmacia Biotech, Piscataway Township, NJ, USA).

A standard curve with a control antioxidant, ascorbic acid (vitamin C) was performed at 10, 5, 1, 0.5, 0.1, 0.05 and 0.01 μg ml^−1^ concentrations in methanol. The detection threshold was established at 0.5 μg ml^−1^ of vitamin C, as lower concentrations of vitamin C did not change DPPH absorbance (data not shown).

DPPH scavenging ability was quantified by measuring the decrease in the absorbance of this compound at 517 nm, and the percentage of scavenged DPPH was calculated using the following formula:

$$\%\;\text{DPPH}=\left(1-\frac{\text{Abs}\;517\;\text{Extract}}{\text{Abs}\;517\;\text{Control}}\right)\times 100.$$

### *In vivo* oxidative stress assays with *C. elegans*

Wild‐type *C. elegans* strain N2 (Bristol, UK) was routinely propagated at 20°C on Nematode Growth Medium (NGM) plates supplemented with *E. coli* strain OP50 as the regular food source.

Nematodes were synchronized by isolating eggs from gravid adults at 20°C. Synchronization was performed on NGM plates with different treatments: *E. coli* OP50 was supplied as a negative control; *E. coli* OP50 plus vitamin C (vitC) at 10 μg ml^−1^ as a positive control; and, finally, *E. coli* OP50 plus one of the selected isolates was used in order to test the effect of administrating the selected strains. Duplicates were performed for every condition. Bacterial strains were grown overnight in liquid LB medium at 28°C and 11,300 *g*. Then, OD_600_ was adjusted to 30 and 50 μl of the bacterial suspension were added to the plates.

The synchronized worms were incubated for 3 days on the previously described plates, until reaching young adult stage. Then, young adult worms were selected for each treatment (*n *=* *50) and incubated at 20°C on the corresponding treatment, until reaching 5‐day adult stage. The selected worms were then transferred to plates containing basal medium supplemented with 2 mM H_2_O_2_ and incubated for 5 h at 20°C. After incubation, survival rates for each condition (negative control, positive control and bacteria‐fed worms) were recorded by manually counting the number of living versus dead worms.

## Conflict of interest

The authors declare no conflict of interest.

## Author contributions

MP conceived the work. MP, KT and EMM collected the samples. EMM, KT and ÀVV performed the culture‐based characterization, and KT carried out the bioinformatic analysis. All authors (MP, KT, ÀVV, EMM and JP) analysed the results, wrote and approved the manuscript.

## Supporting information


**Fig. S1.** Rarefaction curve at OTU level.

**Fig. S2.** Main bacterial (A), archaeal (B) and eukaryotic (C) groups identified in the sample obtained from Vinaròs and analysed through metagenomics sequencing.

**Fig. S3.** Main bacterial (A), archaeal (B) and eukaryotic (C) groups identified in the sample obtained from Cullera and analysed through metagenomics sequencing.

**Fig. S4.** Main bacterial (A), archaeal (B) and eukaryotic (C) groups identified in the sample obtained from Dénia and analysed through metagenomics sequencing.

**Table S1.** Top 30 most significant genera and *P*‐values for the One‐Way ANOVA statistical analysis of their distributions among the three sampled locations. Global *P*‐values and *P*‐values for the comparison by pairs is shown. Significant results are marked by an asterisk.
**Table S2.** List of the strains identified in the collection, with the closest type strain, accession number, ID percentage and the GenBank accession number for the 16S rRNA sequences. The identification code of the strains corresponds to the location from which it was isolated (V: Vinaròs, C: Cullera, D: Dènia), the sample type (R: rock, M: marine water) and a number.
