# Supplementary figures and images for: Microbial communities of the Mediterranean rocky shore: ecology and biotechnological potential of the sea‐land transition
Source: Microb Biotechnol. 2019 Sep 28;12(6):1359–70. doi: 10.1111/1751-7915.13475 (PMC6801134; doi:10.1111/1751-7915.13475)

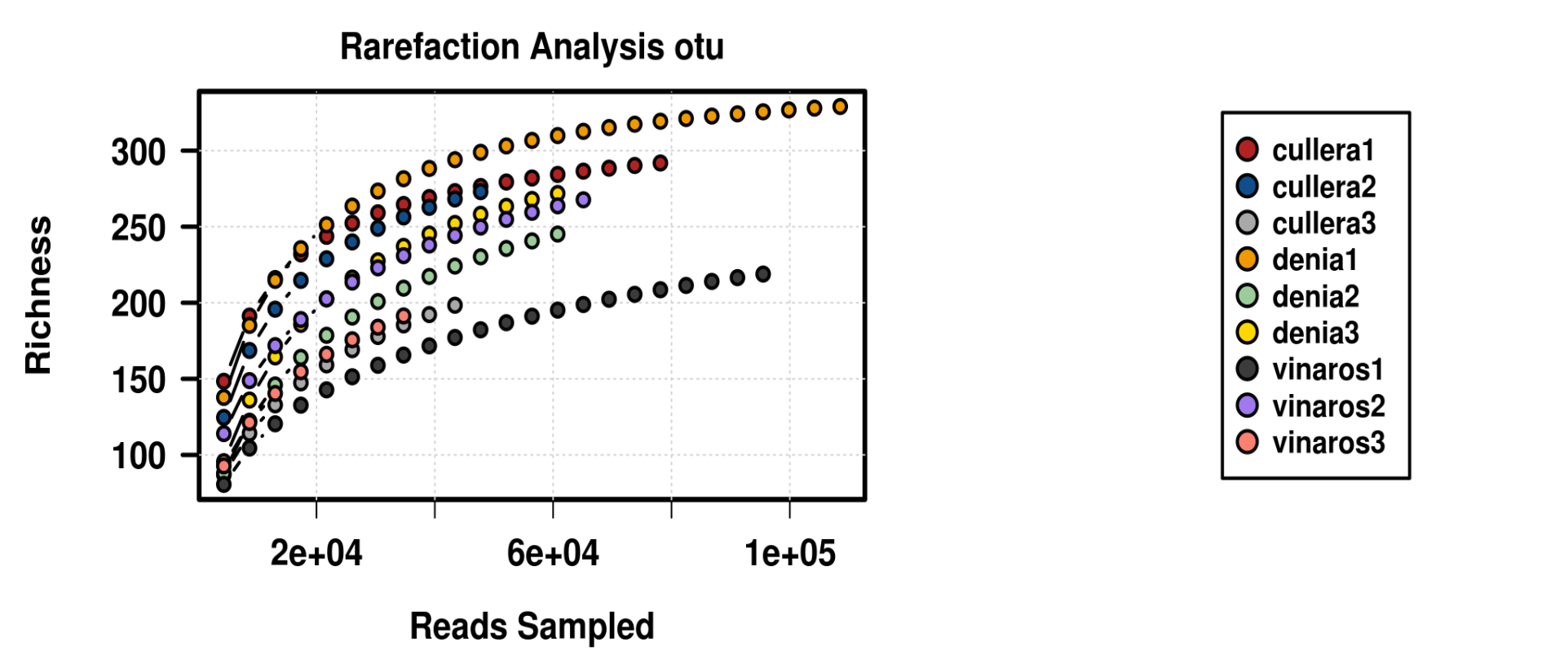

Supplement: Supplementary file 1 — Fig. S1. Rarefaction curve at OTU level. [file MBT2-12-1359-s001.tiff]

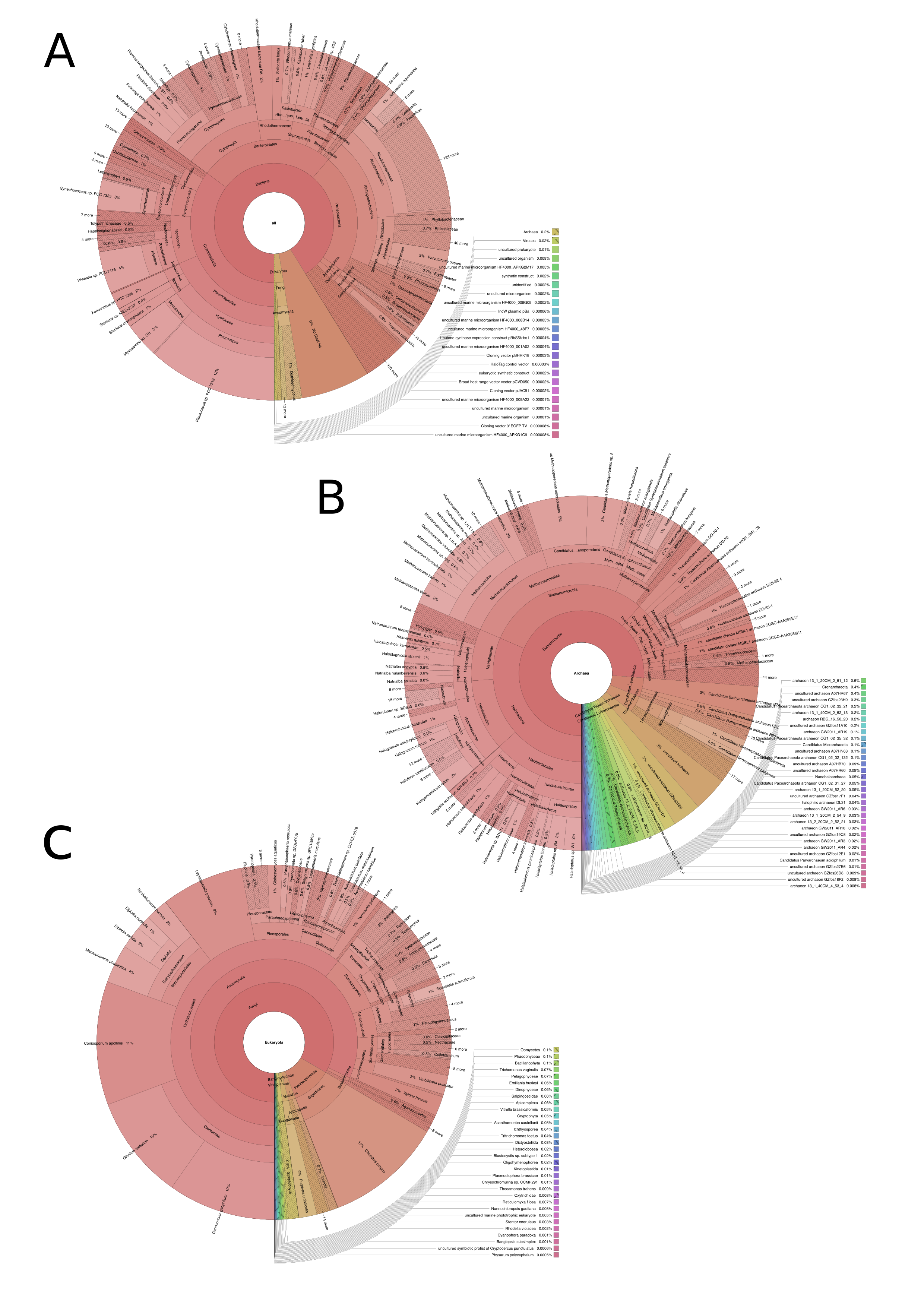

Supplement: Supplementary file 2 — Fig. S2. Main bacterial (A), archaeal (B) and eukaryotic (C) groups identified in the sample obtained from Vinaròs and analysed through metagenomics sequencing. [file MBT2-12-1359-s002.tiff]

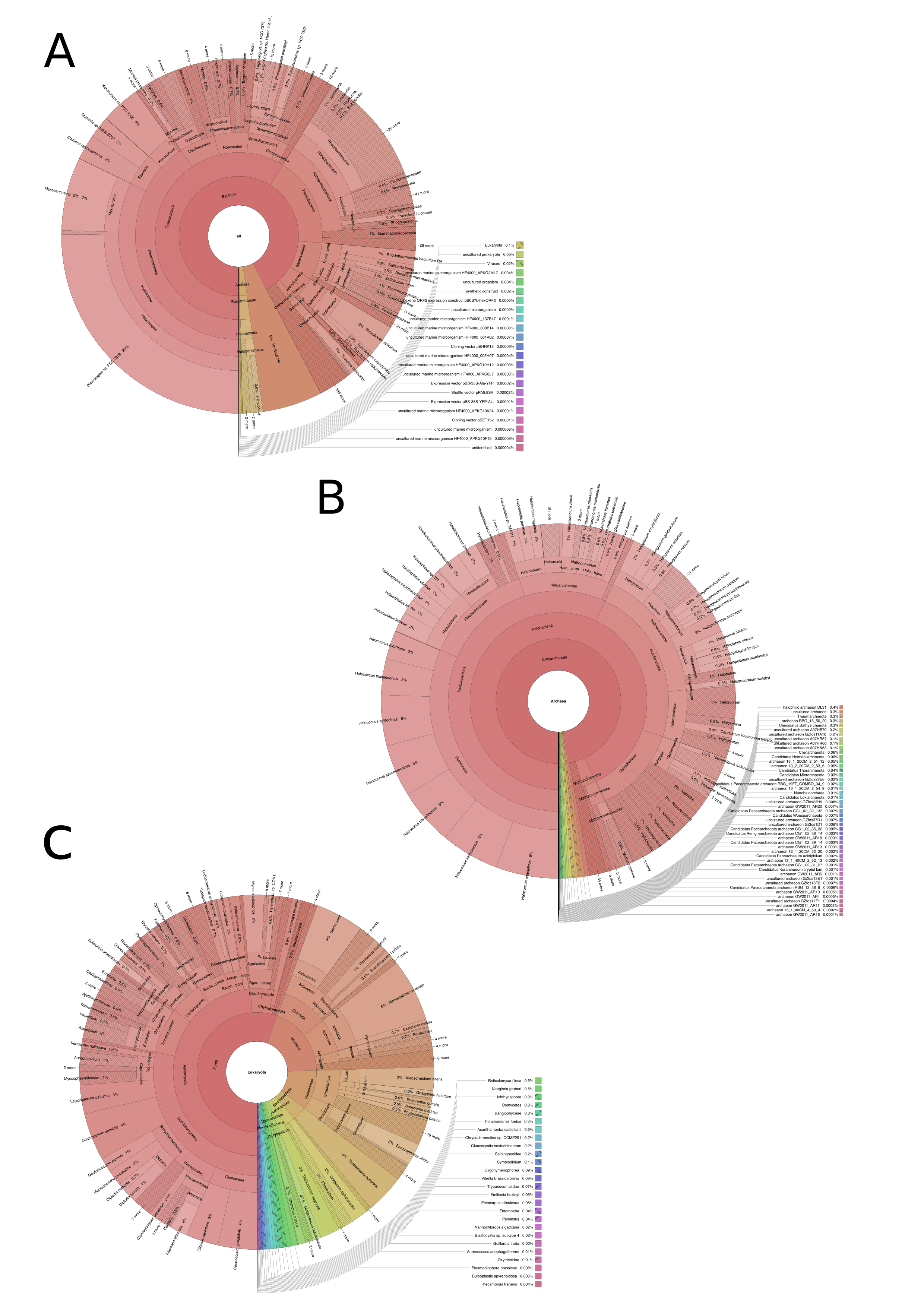

Supplement: Supplementary file 3 — Fig. S3. Main bacterial (A), archaeal (B) and eukaryotic (C) groups identified in the sample obtained from Cullera and analysed through metagenomics sequencing. [file MBT2-12-1359-s003.tiff]

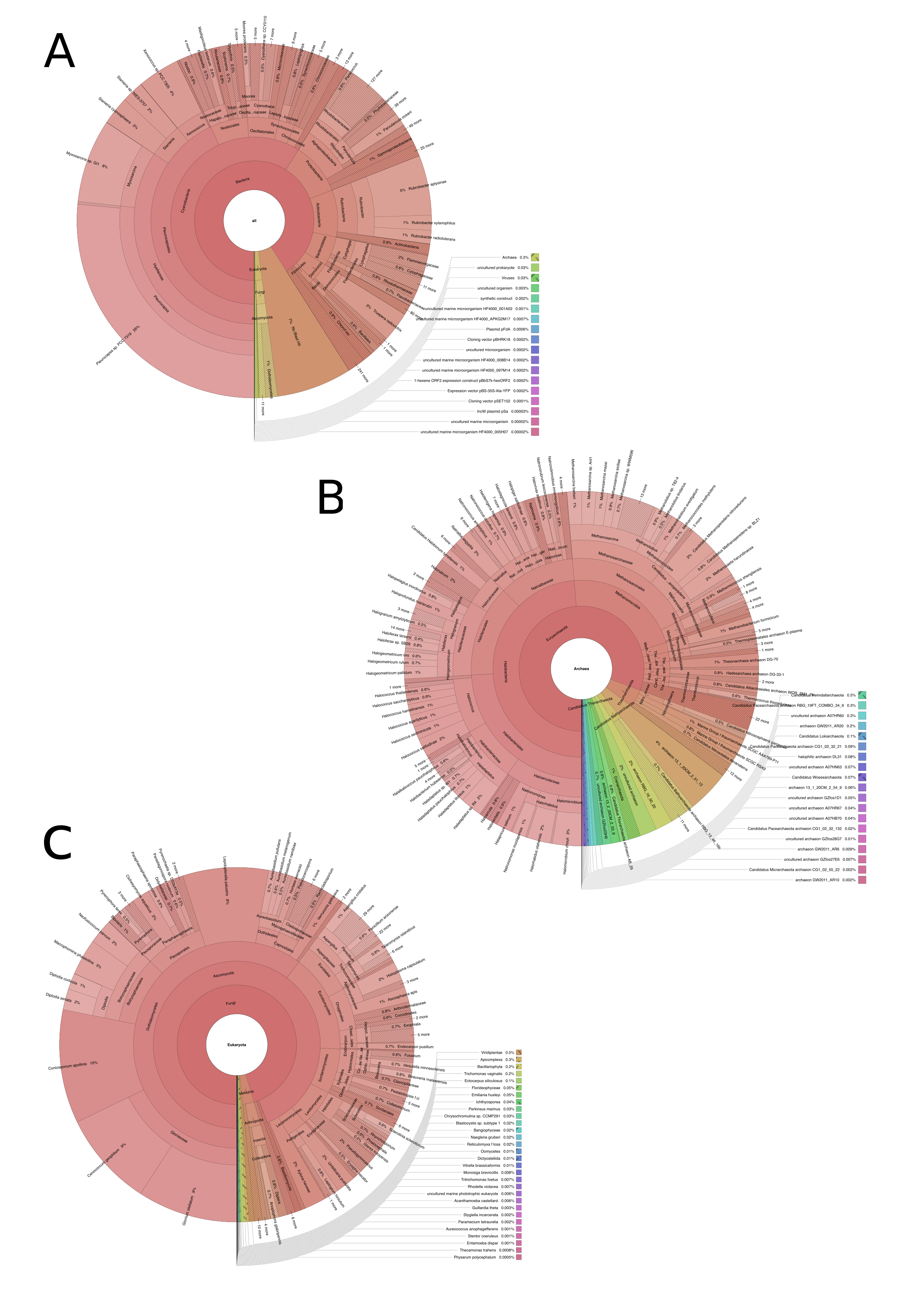

Supplement: Supplementary file 4 — Fig. S4. Main bacterial (A), archaeal (B) and eukaryotic (C) groups identified in the sample obtained from Dénia and analysed through metagenomics sequencing. [file MBT2-12-1359-s004.tiff]
