# Supplementary material for: Microbial communities of the Mediterranean rocky shore: ecology and biotechnological potential of the sea‐land transition
Source: Microb Biotechnol. 2019 Sep 28;12(6):1359–70. doi: 10.1111/1751-7915.13475 (PMC6801134; doi:10.1111/1751-7915.13475)
Supplement: Supplementary file 5 — Table S1. Top 30 most significant genera and P‐values for the One‐Way ANOVA statistical analysis of their distributions among the three sampled locations. Global P‐values and P‐values for the comparison by pairs is shown. Significant results are marked by an asterisk. Table S2. List of the strains identified in the collection, with the closest type strain, accession number, ID percentage and the GenBank accession number for the 16S rRNA sequences. The identification code of the strains corresponds to the location from which it was isolated (V: Vinaròs, C: Cullera, D: Dènia), the sample type (R: rock, M: marine water) and a number. [file MBT2-12-1359-s005.pptx]

## Slide 1
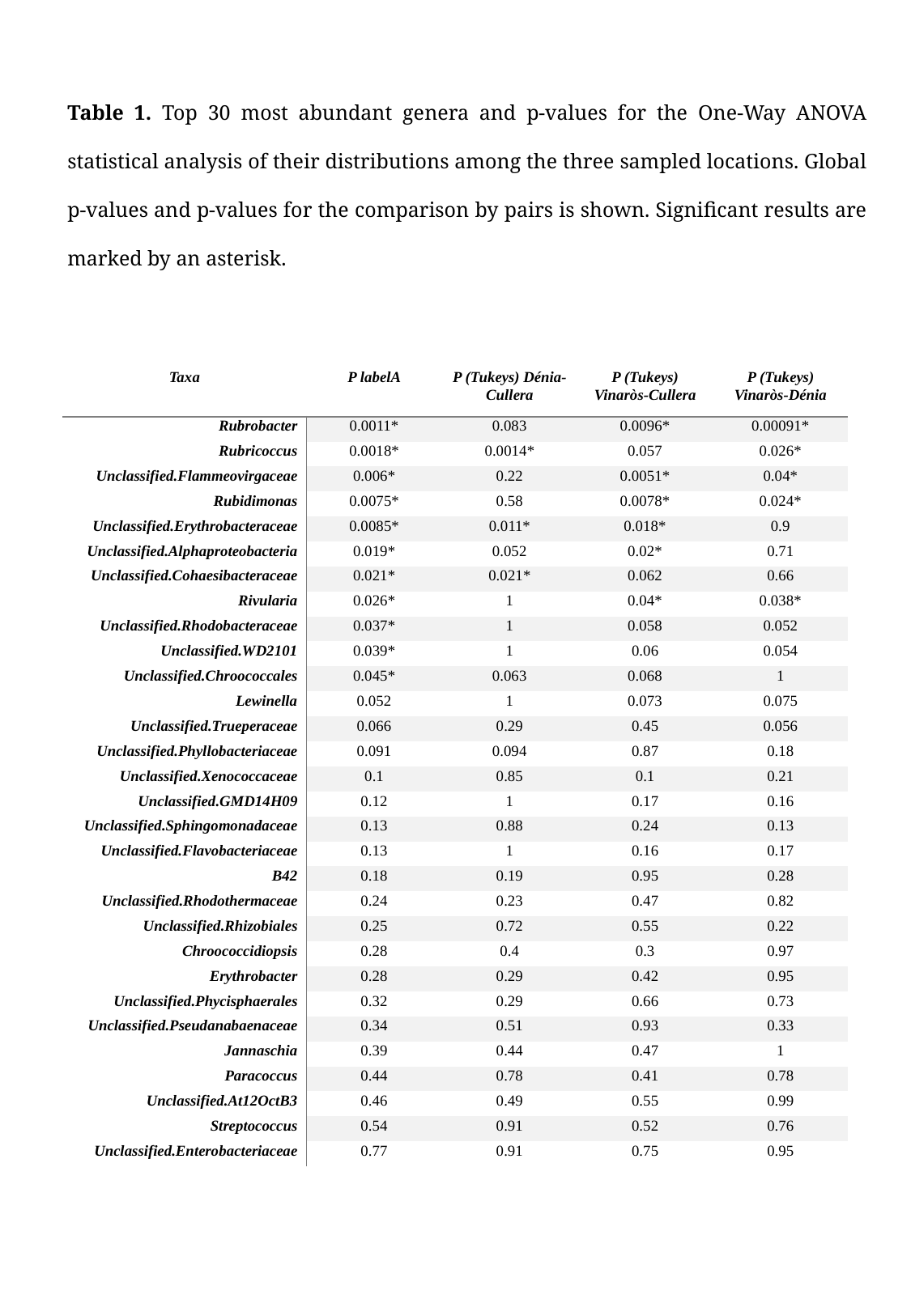

Table 1. Top 30 most abundant genera and p-values for the One-Way ANOVA statistical analysis of their distributions among the three sampled locations. Global p-values and p-values for the comparison by pairs is shown. Significant results are marked by an asterisk.
| Taxa | P labelA | P (Tukeys) Dénia-Cullera | P (Tukeys) Vinaròs-Cullera | P (Tukeys) Vinaròs-Dénia |
| --- | --- | --- | --- | --- |
| Rubrobacter | 0.0011\* | 0.083 | 0.0096\* | 0.00091\* |
| Rubricoccus | 0.0018\* | 0.0014\* | 0.057 | 0.026\* |
| Unclassified.Flammeovirgaceae | 0.006\* | 0.22 | 0.0051\* | 0.04\* |
| Rubidimonas | 0.0075\* | 0.58 | 0.0078\* | 0.024\* |
| Unclassified.Erythrobacteraceae | 0.0085\* | 0.011\* | 0.018\* | 0.9 |
| Unclassified.Alphaproteobacteria | 0.019\* | 0.052 | 0.02\* | 0.71 |
| Unclassified.Cohaesibacteraceae | 0.021\* | 0.021\* | 0.062 | 0.66 |
| Rivularia | 0.026\* | 1 | 0.04\* | 0.038\* |
| Unclassified.Rhodobacteraceae | 0.037\* | 1 | 0.058 | 0.052 |
| Unclassified.WD2101 | 0.039\* | 1 | 0.06 | 0.054 |
| Unclassified.Chroococcales | 0.045\* | 0.063 | 0.068 | 1 |
| Lewinella | 0.052 | 1 | 0.073 | 0.075 |
| Unclassified.Trueperaceae | 0.066 | 0.29 | 0.45 | 0.056 |
| Unclassified.Phyllobacteriaceae | 0.091 | 0.094 | 0.87 | 0.18 |
| Unclassified.Xenococcaceae | 0.1 | 0.85 | 0.1 | 0.21 |
| Unclassified.GMD14H09 | 0.12 | 1 | 0.17 | 0.16 |
| Unclassified.Sphingomonadaceae | 0.13 | 0.88 | 0.24 | 0.13 |
| Unclassified.Flavobacteriaceae | 0.13 | 1 | 0.16 | 0.17 |
| B42 | 0.18 | 0.19 | 0.95 | 0.28 |
| Unclassified.Rhodothermaceae | 0.24 | 0.23 | 0.47 | 0.82 |
| Unclassified.Rhizobiales | 0.25 | 0.72 | 0.55 | 0.22 |
| Chroococcidiopsis | 0.28 | 0.4 | 0.3 | 0.97 |
| Erythrobacter | 0.28 | 0.29 | 0.42 | 0.95 |
| Unclassified.Phycisphaerales | 0.32 | 0.29 | 0.66 | 0.73 |
| Unclassified.Pseudanabaenaceae | 0.34 | 0.51 | 0.93 | 0.33 |
| Jannaschia | 0.39 | 0.44 | 0.47 | 1 |
| Paracoccus | 0.44 | 0.78 | 0.41 | 0.78 |
| Unclassified.At12OctB3 | 0.46 | 0.49 | 0.55 | 0.99 |
| Streptococcus | 0.54 | 0.91 | 0.52 | 0.76 |
| Unclassified.Enterobacteriaceae | 0.77 | 0.91 | 0.75 | 0.95 |

## Slide 2
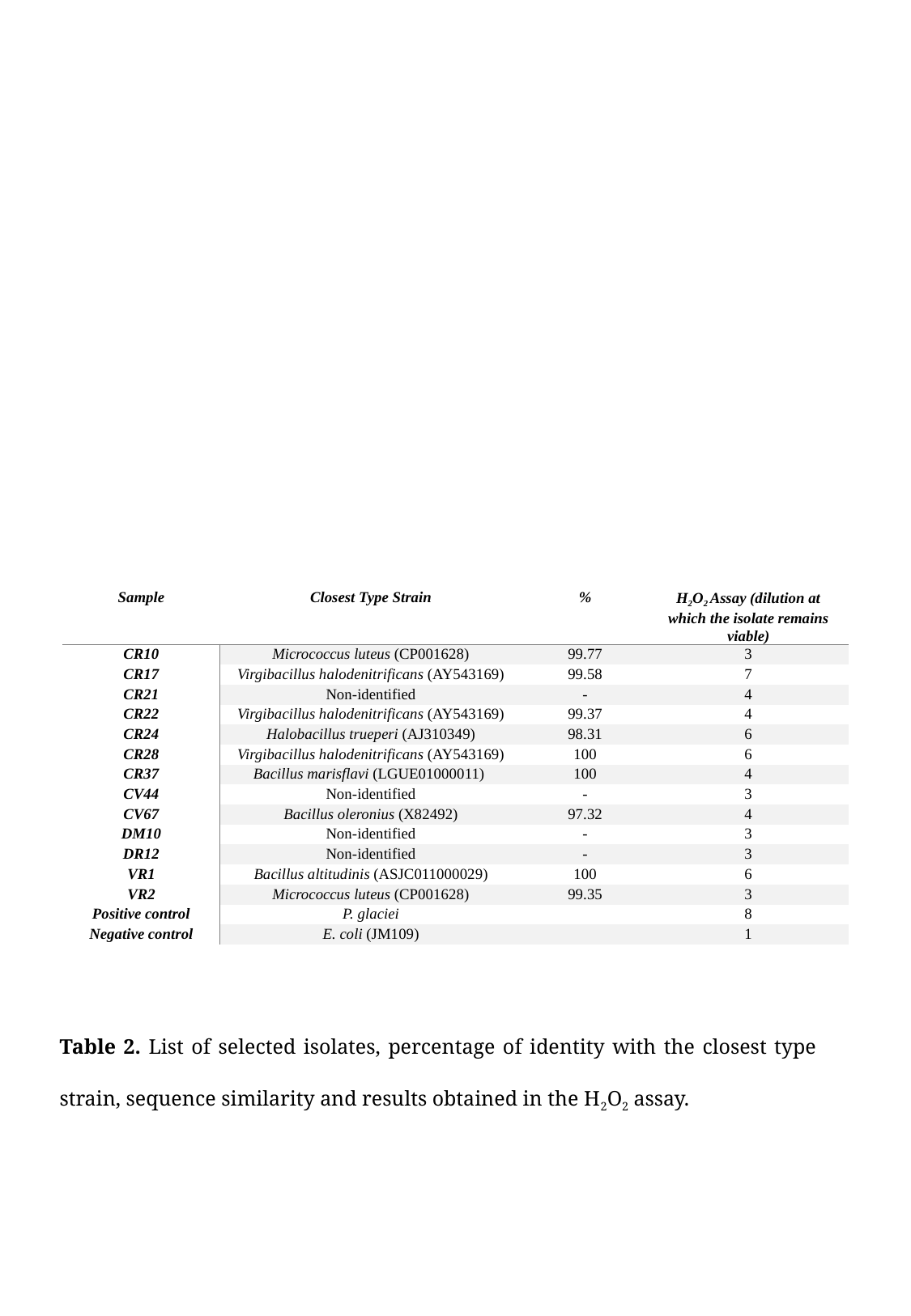

| Sample | Closest Type Strain | % | H2O2 Assay (dilution at which the isolate remains viable) |
| --- | --- | --- | --- |
| CR10 | Micrococcus luteus (CP001628) | 99.77 | 3 |
| CR17 | Virgibacillus halodenitrificans (AY543169) | 99.58 | 7 |
| CR21 | Non-identified | - | 4 |
| CR22 | Virgibacillus halodenitrificans (AY543169) | 99.37 | 4 |
| CR24 | Halobacillus trueperi (AJ310349) | 98.31 | 6 |
| CR28 | Virgibacillus halodenitrificans (AY543169) | 100 | 6 |
| CR37 | Bacillus marisflavi (LGUE01000011) | 100 | 4 |
| CV44 | Non-identified | - | 3 |
| CV67 | Bacillus oleronius (X82492) | 97.32 | 4 |
| DM10 | Non-identified | - | 3 |
| DR12 | Non-identified | - | 3 |
| VR1 | Bacillus altitudinis (ASJC011000029) | 100 | 6 |
| VR2 | Micrococcus luteus (CP001628) | 99.35 | 3 |
| Positive control | P. glaciei | | 8 |
| Negative control | E. coli (JM109) | | 1 |
Table 2. List of selected isolates, percentage of identity with the closest type strain, sequence similarity and results obtained in the H2O2 assay.

## Slide 3
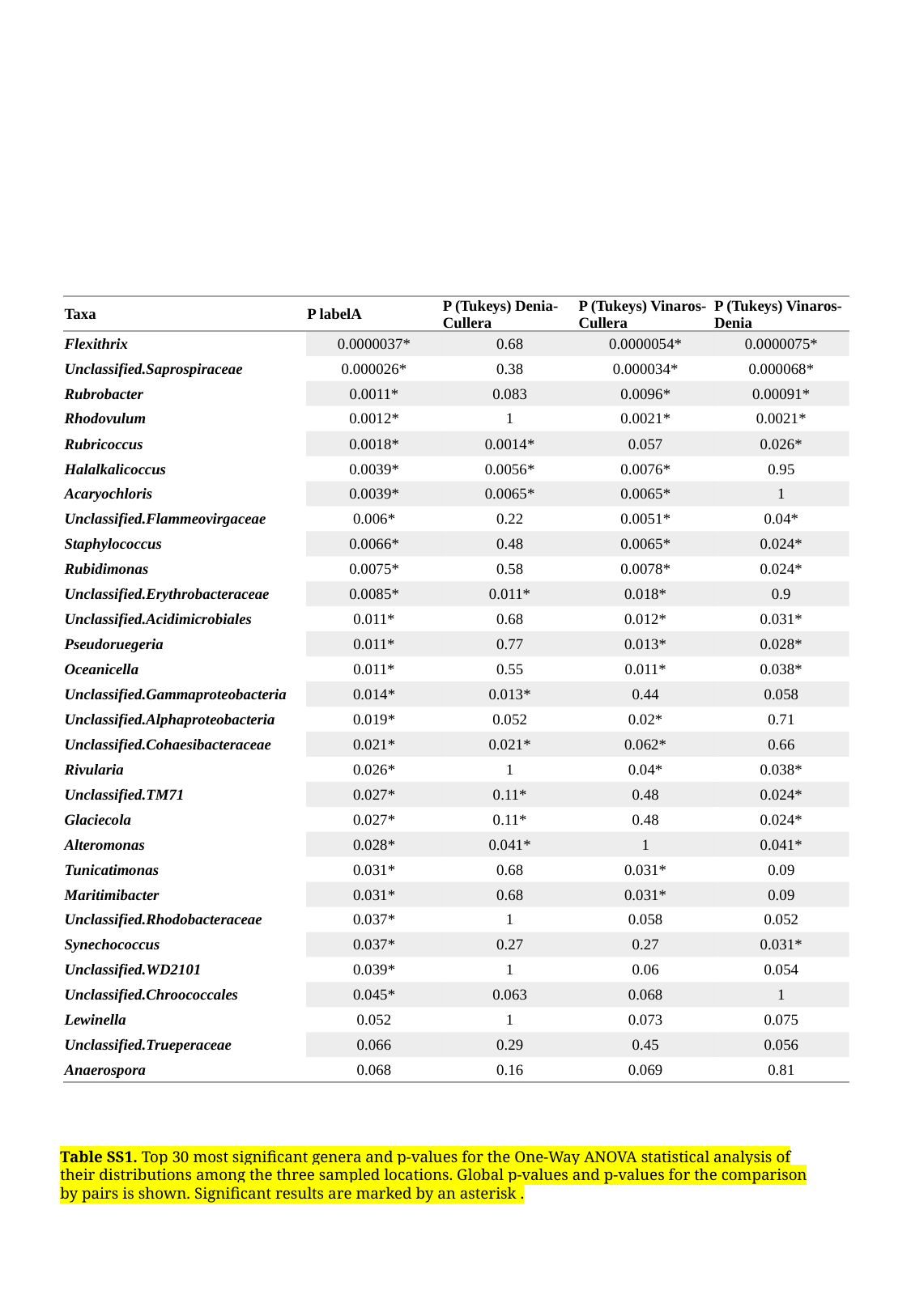

| Taxa | P labelA | P (Tukeys) Denia-Cullera | P (Tukeys) Vinaros-Cullera | P (Tukeys) Vinaros-Denia |
| --- | --- | --- | --- | --- |
| Flexithrix | 0.0000037\* | 0.68 | 0.0000054\* | 0.0000075\* |
| Unclassified.Saprospiraceae | 0.000026\* | 0.38 | 0.000034\* | 0.000068\* |
| Rubrobacter | 0.0011\* | 0.083 | 0.0096\* | 0.00091\* |
| Rhodovulum | 0.0012\* | 1 | 0.0021\* | 0.0021\* |
| Rubricoccus | 0.0018\* | 0.0014\* | 0.057 | 0.026\* |
| Halalkalicoccus | 0.0039\* | 0.0056\* | 0.0076\* | 0.95 |
| Acaryochloris | 0.0039\* | 0.0065\* | 0.0065\* | 1 |
| Unclassified.Flammeovirgaceae | 0.006\* | 0.22 | 0.0051\* | 0.04\* |
| Staphylococcus | 0.0066\* | 0.48 | 0.0065\* | 0.024\* |
| Rubidimonas | 0.0075\* | 0.58 | 0.0078\* | 0.024\* |
| Unclassified.Erythrobacteraceae | 0.0085\* | 0.011\* | 0.018\* | 0.9 |
| Unclassified.Acidimicrobiales | 0.011\* | 0.68 | 0.012\* | 0.031\* |
| Pseudoruegeria | 0.011\* | 0.77 | 0.013\* | 0.028\* |
| Oceanicella | 0.011\* | 0.55 | 0.011\* | 0.038\* |
| Unclassified.Gammaproteobacteria | 0.014\* | 0.013\* | 0.44 | 0.058 |
| Unclassified.Alphaproteobacteria | 0.019\* | 0.052 | 0.02\* | 0.71 |
| Unclassified.Cohaesibacteraceae | 0.021\* | 0.021\* | 0.062\* | 0.66 |
| Rivularia | 0.026\* | 1 | 0.04\* | 0.038\* |
| Unclassified.TM71 | 0.027\* | 0.11\* | 0.48 | 0.024\* |
| Glaciecola | 0.027\* | 0.11\* | 0.48 | 0.024\* |
| Alteromonas | 0.028\* | 0.041\* | 1 | 0.041\* |
| Tunicatimonas | 0.031\* | 0.68 | 0.031\* | 0.09 |
| Maritimibacter | 0.031\* | 0.68 | 0.031\* | 0.09 |
| Unclassified.Rhodobacteraceae | 0.037\* | 1 | 0.058 | 0.052 |
| Synechococcus | 0.037\* | 0.27 | 0.27 | 0.031\* |
| Unclassified.WD2101 | 0.039\* | 1 | 0.06 | 0.054 |
| Unclassified.Chroococcales | 0.045\* | 0.063 | 0.068 | 1 |
| Lewinella | 0.052 | 1 | 0.073 | 0.075 |
| Unclassified.Trueperaceae | 0.066 | 0.29 | 0.45 | 0.056 |
| Anaerospora | 0.068 | 0.16 | 0.069 | 0.81 |
Table SS1. Top 30 most significant genera and p-values for the One-Way ANOVA statistical analysis of their distributions among the three sampled locations. Global p-values and p-values for the comparison by pairs is shown. Significant results are marked by an asterisk .

## Slide 4
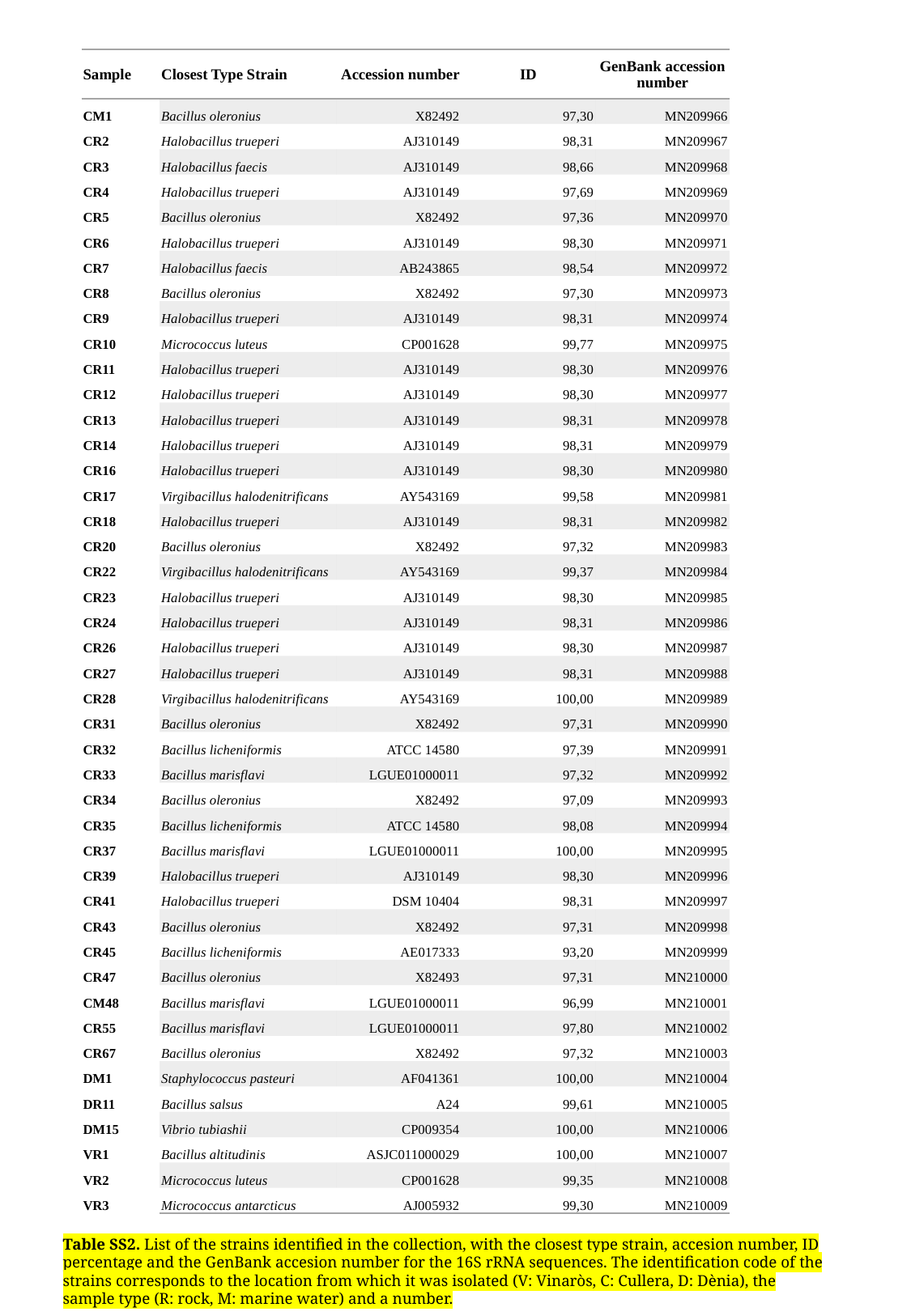

| Sample | Closest Type Strain | Accession number | ID | GenBank accession number |
| --- | --- | --- | --- | --- |
| CM1 | Bacillus oleronius | X82492 | 97,30 | MN209966 |
| CR2 | Halobacillus trueperi | AJ310149 | 98,31 | MN209967 |
| CR3 | Halobacillus faecis | AJ310149 | 98,66 | MN209968 |
| CR4 | Halobacillus trueperi | AJ310149 | 97,69 | MN209969 |
| CR5 | Bacillus oleronius | X82492 | 97,36 | MN209970 |
| CR6 | Halobacillus trueperi | AJ310149 | 98,30 | MN209971 |
| CR7 | Halobacillus faecis | AB243865 | 98,54 | MN209972 |
| CR8 | Bacillus oleronius | X82492 | 97,30 | MN209973 |
| CR9 | Halobacillus trueperi | AJ310149 | 98,31 | MN209974 |
| CR10 | Micrococcus luteus | CP001628 | 99,77 | MN209975 |
| CR11 | Halobacillus trueperi | AJ310149 | 98,30 | MN209976 |
| CR12 | Halobacillus trueperi | AJ310149 | 98,30 | MN209977 |
| CR13 | Halobacillus trueperi | AJ310149 | 98,31 | MN209978 |
| CR14 | Halobacillus trueperi | AJ310149 | 98,31 | MN209979 |
| CR16 | Halobacillus trueperi | AJ310149 | 98,30 | MN209980 |
| CR17 | Virgibacillus halodenitrificans | AY543169 | 99,58 | MN209981 |
| CR18 | Halobacillus trueperi | AJ310149 | 98,31 | MN209982 |
| CR20 | Bacillus oleronius | X82492 | 97,32 | MN209983 |
| CR22 | Virgibacillus halodenitrificans | AY543169 | 99,37 | MN209984 |
| CR23 | Halobacillus trueperi | AJ310149 | 98,30 | MN209985 |
| CR24 | Halobacillus trueperi | AJ310149 | 98,31 | MN209986 |
| CR26 | Halobacillus trueperi | AJ310149 | 98,30 | MN209987 |
| CR27 | Halobacillus trueperi | AJ310149 | 98,31 | MN209988 |
| CR28 | Virgibacillus halodenitrificans | AY543169 | 100,00 | MN209989 |
| CR31 | Bacillus oleronius | X82492 | 97,31 | MN209990 |
| CR32 | Bacillus licheniformis | ATCC 14580 | 97,39 | MN209991 |
| CR33 | Bacillus marisflavi | LGUE01000011 | 97,32 | MN209992 |
| CR34 | Bacillus oleronius | X82492 | 97,09 | MN209993 |
| CR35 | Bacillus licheniformis | ATCC 14580 | 98,08 | MN209994 |
| CR37 | Bacillus marisflavi | LGUE01000011 | 100,00 | MN209995 |
| CR39 | Halobacillus trueperi | AJ310149 | 98,30 | MN209996 |
| CR41 | Halobacillus trueperi | DSM 10404 | 98,31 | MN209997 |
| CR43 | Bacillus oleronius | X82492 | 97,31 | MN209998 |
| CR45 | Bacillus licheniformis | AE017333 | 93,20 | MN209999 |
| CR47 | Bacillus oleronius | X82493 | 97,31 | MN210000 |
| CM48 | Bacillus marisflavi | LGUE01000011 | 96,99 | MN210001 |
| CR55 | Bacillus marisflavi | LGUE01000011 | 97,80 | MN210002 |
| CR67 | Bacillus oleronius | X82492 | 97,32 | MN210003 |
| DM1 | Staphylococcus pasteuri | AF041361 | 100,00 | MN210004 |
| DR11 | Bacillus salsus | A24 | 99,61 | MN210005 |
| DM15 | Vibrio tubiashii | CP009354 | 100,00 | MN210006 |
| VR1 | Bacillus altitudinis | ASJC011000029 | 100,00 | MN210007 |
| VR2 | Micrococcus luteus | CP001628 | 99,35 | MN210008 |
| VR3 | Micrococcus antarcticus | AJ005932 | 99,30 | MN210009 |
Table SS2. List of the strains identified in the collection, with the closest type strain, accesion number, ID percentage and the GenBank accesion number for the 16S rRNA sequences. The identification code of the strains corresponds to the location from which it was isolated (V: Vinaròs, C: Cullera, D: Dènia), the sample type (R: rock, M: marine water) and a number.
